# Dimensional effects of surface morphology and trapped air on mammalian cell adhesion to special wetting surfaces

**DOI:** 10.1093/rb/rbaf021

**Published:** 2025-04-01

**Authors:** Zhiwei Chen, Yun Yang, Shaohua Xu, Zhenyu Shen, Yijian Tang, Yisheng Lin, Qiaoling Huang

**Affiliations:** Research Institute for Biomimetics and Soft Matter, Fujian Provincial Key Laboratory for Soft Functional Materials Research, Department of Physics, College of Physical Science and Technology, Xiamen University, Xiamen 361005, China; Research Institute for Biomimetics and Soft Matter, Fujian Provincial Key Laboratory for Soft Functional Materials Research, Department of Physics, College of Physical Science and Technology, Xiamen University, Xiamen 361005, China; Jiujiang Research Institute of Xiamen University, Jiujiang 332000, China; Research Institute for Biomimetics and Soft Matter, Fujian Provincial Key Laboratory for Soft Functional Materials Research, Department of Physics, College of Physical Science and Technology, Xiamen University, Xiamen 361005, China; Jiujiang Research Institute of Xiamen University, Jiujiang 332000, China; Research Institute for Biomimetics and Soft Matter, Fujian Provincial Key Laboratory for Soft Functional Materials Research, Department of Physics, College of Physical Science and Technology, Xiamen University, Xiamen 361005, China; Research Institute for Biomimetics and Soft Matter, Fujian Provincial Key Laboratory for Soft Functional Materials Research, Department of Physics, College of Physical Science and Technology, Xiamen University, Xiamen 361005, China; Research Institute for Biomimetics and Soft Matter, Fujian Provincial Key Laboratory for Soft Functional Materials Research, Department of Physics, College of Physical Science and Technology, Xiamen University, Xiamen 361005, China; Research Institute for Biomimetics and Soft Matter, Fujian Provincial Key Laboratory for Soft Functional Materials Research, Department of Physics, College of Physical Science and Technology, Xiamen University, Xiamen 361005, China; Jiujiang Research Institute of Xiamen University, Jiujiang 332000, China

**Keywords:** superhydrophobic materials, mammalian cell adhesion, trapped air, dimensional effects

## Abstract

Materials with special wettability have broad biomedical applications, including the control of mammalian cell adhesion and inhibiting biofilm formation. However, limited understanding of mammalian cellular responses to superhydrophobic materials with trapped air restricts their clinical applications. In this study, we fabricated materials with varied nanostructures and wettability, and systematically compared short-term mammalian cellular responses in the presence and absence of trapped air. Our results show that small nanostructures generate small, often invisible air bubbles at the solid–liquid interface when in contact with mammalian cell suspensions. In the presence of these small bubbles, the number of adhered cells was comparable to both the same sample without trapped air and its hydrophilic counterpart, contradicting the intuitive expectations that trapped air would reduce cell adhesion. In contrast, larger nanostructures resulted in visible, hundred-micron-sized air bubbles, which significantly inhibited cell adhesion. This effect was evident when comparing the same superhydrophobic sample with and without trapped air, as well as against hydrophilic counterparts with the same morphology. Further tracking of large air bubbles on the hydrophobic materials revealed that no cells adhered to the areas occupied by hundred-micron-sized air bubbles, while more cells accumulated at the solid–liquid–gas triple line. Hence, this work deepens the understanding of cellular responses to superhydrophobic materials, revealing that material structure size influences the size of trapped air and subsequently dominates cell adhesion.

## Graphical abstract

**Figure:**
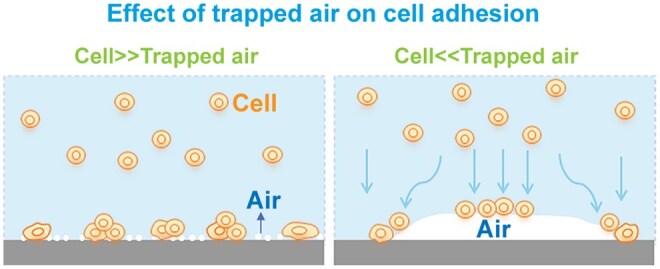

## Introduction

Upon implantation, a series of complex biological process happens on biomaterials surfaces [1], including small molecules adsorption, protein adsorption [2–4], mammalian cell adhesion [5], bacteria adhesion [6, 7], etc. These interfacial interactions are governed not only by the biological environment, but also by the physical and chemical properties of the material surface, such as surface composition [8], morphology [9, 10], wettability [11–13] and surface energy [14, 15]. Among these, wettability is one of the most distinct properties that dominate biological responses to biomaterials [16, 17].

Superhydrophobic materials are those that possess an exceptionally strong water-repellent property, causing water droplets to form spherical shapes with a contact angle greater than 150°. Due to their novel wettability, superhydrophobic materials display exceptional properties, such as self-cleaning [18], anti-fouling [19, 20], water collection [21, 22], corrosion resistance [23] and ice resistance [24, 25], which have broad application prospects across diverse fields. In the biomedical field, superhydrophobic materials also exhibit excellent anti-adhesion properties, such as regulating protein adsorption [26, 27] and mammalian cell adhesion [28, 29], anticoagulation [23, 30–33], inhibiting bacteria [34–37], drug delivery [38, 39] and high-throughput cell screening [40].

The repellent effect of superhydrophobic materials is often attributed to the air trapped between the material surface and liquid, which can inhibit the intimate interaction with the surface. This trapped air is thought to reduce mammalian cell adhesion. Indeed, many studies have shown that superhydrophobic materials reduce mammalian cell adhesion, typically ascribed to the trapped air. For example, Meng *et al.* [41] showed that nonwetted surfaces are cell-repellent, whereas cells can attach on the wetted surfaces. They ascribed this to the trapped air, which hinders the contact of cells with nonwetted surfaces. However, conflicting results have shown that superhydrophobic materials can enhance mammalian cell adhesion [42]. Previously, we removed trapped air by ultrasonication and directly compared short-term mammalian cell adhesion (2 h) on the same titanium nanopores, in the presence and absence of trapped air [43]. Interestingly, our results showed no discernable difference in the number of adherent cells with and without trapped air. This raises the question: what is the precise role of trapped air in cell adhesion?

From a material properties perspective, although superhydrophobic materials share similar wettability characteristics, they can differ significantly in surface morphology, chemistry and charge. These variations may influence the size and quantity of air trapped at the solid–liquid interface, which in turn could affect cell adhesion. However, no prior studies have isolated the effects of surface morphology from those of trapped air. Specifically, it remains unclear whether the surface structure and trapped air influence cell response independently or whether these factors interact synergistically.

To address this knowledge gap, we first designed four hydrophobic TiO_2_ nanomaterials with contact angle exceeding 150°, featuring different morphologies and air content, to explore how material morphology affects initial adhesion. To investigate the role of trapped air, we removed it and compared cell responses in the presence and absence of trapped air at the solid–liquid interface of the materials. Additionally, we designed transparent superhydrophobic PDSM with micro-nanostructures to track visible millimeter-sized air bubbles and observe cell behavior around them.

## Materials and methods

### Preparation of TiO_2_ samples

TiO_2_ nanomaterials were fabricated via electro-anodization. Briefly, titanium foils were ultrasonically cleaned in acetone, ethanol and deionized water for 15 min each, then air-dried at room temperature. The titanium foil was immersed in a 0.5 wt% HF solution and used as the working electrode, with a platinum sheet serving as the counter electrode. TiO_2_ nanostructures with various morphologies were prepared by adjusting the applied voltage to 3, 5, 20 and 30 V. To achieve hydrophobicity, the TiO_2_ nanostructures were silanized by immersing the electro-anodized titanium foils in a 1 w/v% 1H,1H,2H,2H-perfluorooctyltriethoxysilane solution in methanol for 1 h, followed by curing at 140°C for 1 h.

### Fabrication of transparent superhydrophobic PDMS materials

A four-inch silicon wafer was ultrasonically cleaned in acetone, ethanol and deionized water for 30 min, then dried with nitrogen and placed in a drying box to remove excess water vapor. The dried silicon wafer was placed in a spin coater, where photoresist (AZ5214e) was dropped at the center and spin-coated at 3000 rpm for 40 s (with both acceleration and deceleration times set to 3 s) to ensure even coverage of the wafer surface. After homogenization, the wafer was baked at 96°C for 4 min to evaporate the solvent in the photoresist.

Next, a mask with a circular pattern was placed on the silicon wafer, and exposed using a photolithography machine (MA6), transferring the mask pattern onto the photoresist. A developer (tetramethylammonium hydroxide) was then applied to dissolve the photoresist in the exposed areas (with a development time of 1–2 min). After development, the wafer was baked again at 135°C for 10 min to enhance the stability of the photoresist. Using a deep silicon etching system (AMS200), the surface of the silicon wafer was etched to a depth of 16 μm. After etching, the wafer was ultrasonicated in acetone for 15 min to remove the remaining photoresist, resulting in an array of cylindrical grooves on the silicon template surface.

We mixed the PDMS base material and curing agent at a mass ratio of 10:1, weighed 0.5 g of the mixture and added it to 20 ml of n-hexane, then sonicated for 30 min to fully dissolve PDMS. Next, 0.3 g of fumed hydrophobic silica particles were added to the solution and dispersed ultrasonically for 1 h to prepare a silica dispersion. The silica dispersion was spin-coated onto the surface of the PDMS sample at 3000 rpm for 10 s, then dried at 120°C to form a superhydrophobic PDMS sample (MN-PDMS) with a micro-nano composite structure.

### Material characterization

The surface morphology of the sample was characterized using scanning electron microscopy. The wettability of the sample was assessed with a dynamic contact angle measuring instrument (DSA100) from Dataphysics (Germany). The adhesion force on the superhydrophobic sample surface was measured using a surface tensiometer (DCAT11EC, Dataphysics, Germany).

### Removal of trapped air between solid–liquid interface

To remove trapped air from the solid–liquid interface and prevent re-trapping, we fixed a Teflon O-ring on the SHB surface. The samples were immersed in phosphate-buffered saline (PBS) and ultrasonicated for a few seconds. A certain amount of PBS was retained inside the O-ring to prevent air from re-entering the solid–liquid interface.

### Mammalian cell adhesion

Mouse embryonic osteoblasts (MC3T3-E1) were maintained in an incubator at 37°C under a humidified atmosphere with 5% CO_2_. The culture medium consisted of α-MEM medium (HyClone) supplemented with 10% fetal bovine serum (FBS, Gibco) and 1% penicillin–streptomycin (HyClone). Cells suspensions were seeded onto sample surfaces at a density of 50 000 cells/cm^2^. After 2 h of incubation in the incubator, the sample surface was washed with phosphate-buffered saline (PBS), and the number of adherent cells on different samples was measured using the CCK-8 assay (Beyotime, Jiangsu, China).

For fluorescent staining, cells were incubated with 1 μmol/l Calcein-AM solution (Beyotime, Jiangsu, China) for 15 min and then observed under a fluorescence microscope (DMi8, Leica Microsystems, Germany). To track changes in air bubbles, we labeled the samples, and after 2 h of incubation, we observed the cell adhesion at the locations where air was present using a microscope.

### Cell scratching experiment

Cell suspensions were seeded onto sample surfaces at a density of 50 000 cells/cm^2^. After 18 h of culture, a uniform scratch was created using a sterile 10 µl pipette tip. The cells were then incubated in culture medium under standard conditions (37°C, 5% CO_2_) for 6 h to allow migration into the scratched area. To visualize cell movement and wound closure, the cells were stained with 1 μmol/l Calcein-AM solution for 15 min and observed under a fluorescence microscope.

## Results and discussion

### Characterization of TiO_2_ nanostructures

**Figure 1.**
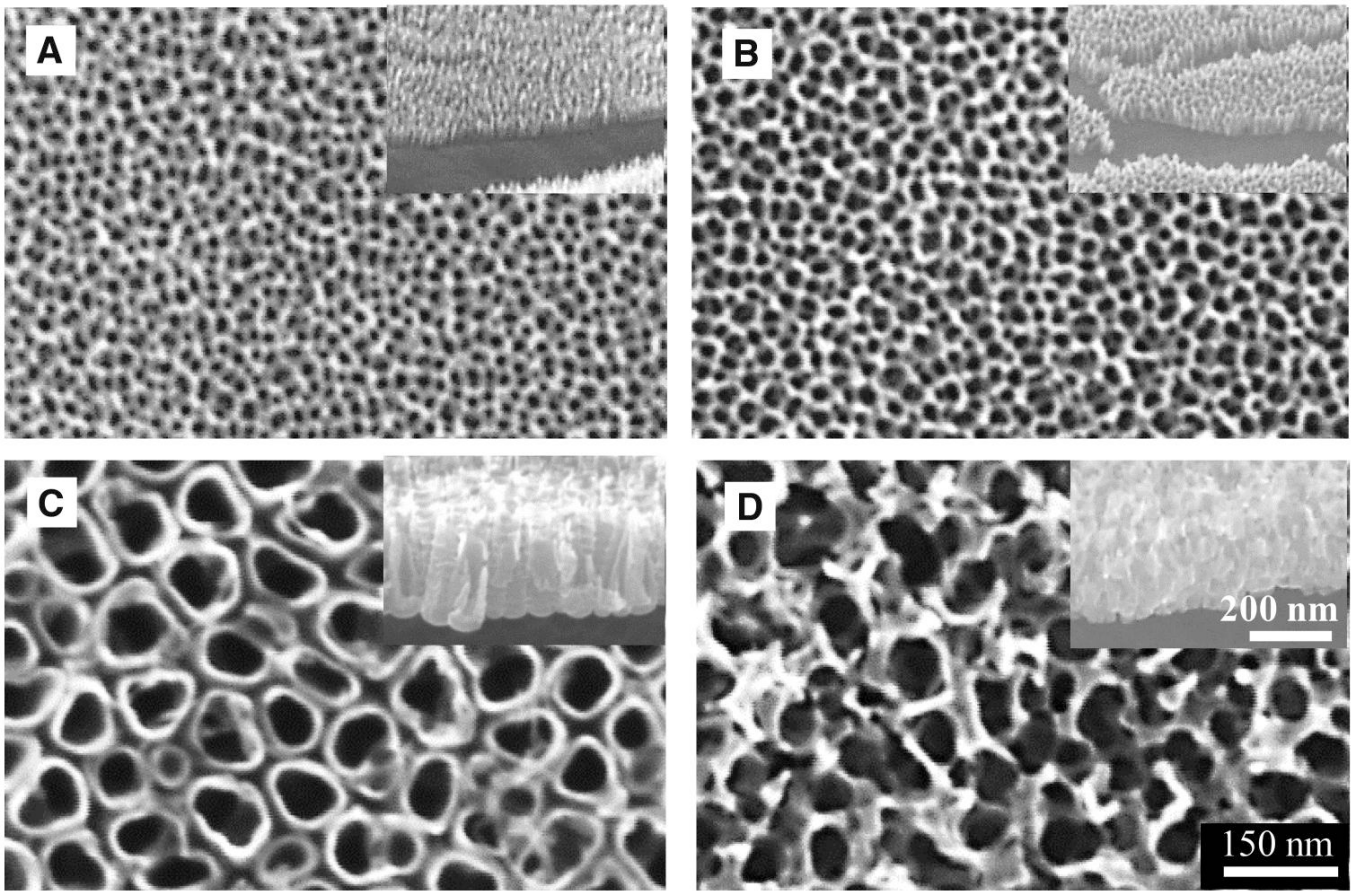
Top view and cross-sectional (insets) SEM images of TiO_2_ nanostructures: (**A**) sNPA, (**B**) NPA, (**C**) NTA and (**D**) NVS.

TiO_2_ nanostructures were fabricated through electro-anodization of titanium. Figure 1 provides an overview of the voltage-dependent nanostructures. At a low voltage of 3 V, the titanium foils oxidize to form small nanopores (sNPA) with a diameter of approximately 20 nm (Figure 1A). As the applied voltage increases to 5 V, the nanopore size expands to ∼30 nm (NPA, Figure 1B). With a further increase in voltage to 20 V, the nanopores gradually enlarge, forming nanotubular structures with a diameter of 100 nm (NTA, Figure 1C). At 30 V, the nanotube structure transforms into sponge-like nano-vesuvianite structures (NVS, Figure 1D). Additionally, as the voltage increases, the thickness of the TiO_2_ films progressively grows from 90 to 100 nm, 300 nm and finally 350 nm.

**Figure 2.**
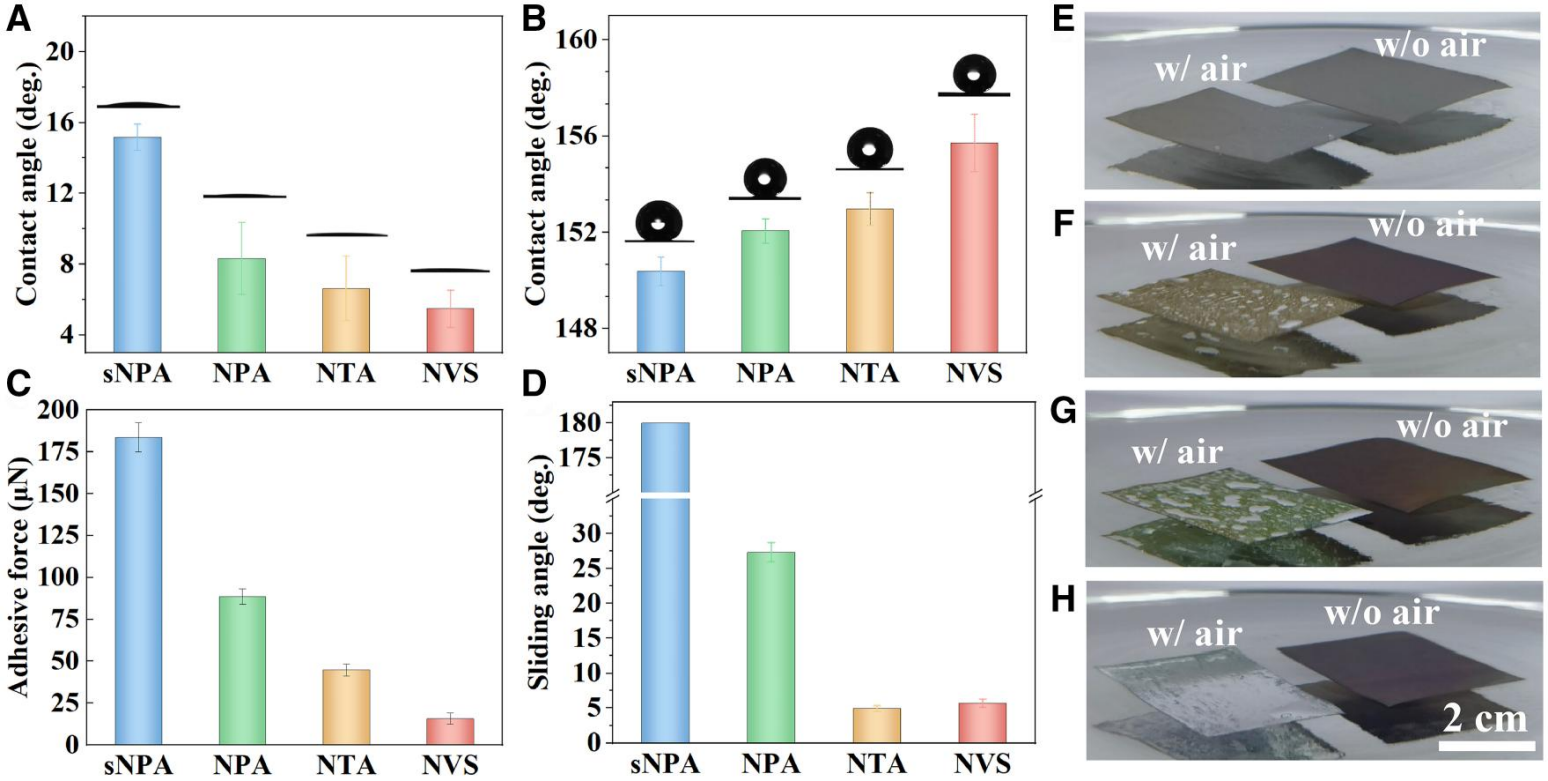
Static water contact angles of various TiO_2_ nanostructures before (**A**) and after (**B**) silanization. Adhesive force (**C**) and sliding angle (**D**) of deionized water on different silanized TiO_2_ nanomaterials. Optical images of silanized materials immersed in water: (**E**) sNPA, (**F**) NPA, (**G**) NTA, (**H**) NVS. ‘w/air’ indicates the sample was not treated with ultrasonication, while ‘w/o air’ indicates the sample was treated by ultrasonication to remove trapped air.

The TiO_2_ nanostructures exhibit a range of wetting behaviors before and after silanization treatment. Initially, all the as-prepared TiO_2_ nanostructures are hydrophilic or superhydrophilic (Figure 2A). However, after silanization, all four samples become hydrophobic or even superhydrophobic, achieving static water contact angles greater than 150° (Figure 2B). For simplicity, we refer to the as-prepared samples as ‘SHL’ and the silanized samples as ‘SHB’.

Interestingly, the adhesive forces (Figure 2C) and sliding angles (Figure 2D) vary significantly among the silanized samples. The silanized SHB-sNPA sample exhibits a high adhesive force of approximately 183 µN and a sliding angle of 180°, indicating water strongly adheres to the surface even when tilted or inverted. This behavior is characteristic of a high-adhesion state, similar to the Wenzel state. Materials with a contact angle higher than 150° and high adhesive force are usually referred to as having the rose petal effect [44].

As the nanostructure size increases, the adhesion force and sliding angle of the silanized samples decrease dramatically. For example, when the nanopore size increases from 20 nm (SHB-sNPA) to 30 nm (SHB-NPA), the adhesive force drops to about 88 µN (Figure 2C), and the sliding angle lowers to around 27° (Figure 2D). In these samples, the high-adhesion property diminishes, allowing water droplets to roll off quickly as the tilt angle increases. In contrast, on the SHB-NTA and SHB-NVS samples, the sliding angles are smaller than 10°, representing a low-adhesion state similar to the Cassie state. Consequently, water droplets roll off easily, illustrating the superhydrophobic nature of the SHB-NTA and SHB-NVS samples. The sharp difference in adhesive force across these samples can be attributed to their distinct nanostructure (Figure 1), despite all four samples undergoing the same silanization treatment. This variation in structure affects the nature of the solid–liquid–gas contact, ultimately influencing the adhesion and wetting behavior [45].

When silanized materials are immersed in water, air can easily become trapped between the solid and liquid interface. Ultrasonication is used to release this trapped air by generating ultrasonic oscillations that dislodge air bubbles from the solid surface. For the SHB-sNPA sample, which appears gray when immersed in water, only a few air bubbles are visible, each in the 100 μm size range (Figure 2E). After ultrasonication, the color of the SHB-sNPA sample remains nearly unchanged, indicating minimal trapped air and suggesting that most regions are likely in the Wenzel state, where the liquid fully penetrates the surface texture.

In contrast, the SHB-NPA sample initially appears light yellow and contains numerous hundred-micron-sized bubbles (Figure 2F). These bubbles disappear after ultrasonication, turning the sample dark brown, which signifies that air has been released from the solid–liquid interface. As the nanostructures increase in size, the SHB-NTA sample displays larger air bubbles on its surface (Figure 2G), reaching the millimeter scale. Further increases in nanostructure size on the superhydrophobic SHB-NVS sample lead to the formation of a continuous air layer, which creates a mirror-like appearance on the surface (Figure 2H). Both the SHB-NTA and SHB-NVS samples turn dark brown after ultrasonication, indicating the removal of most trapped air. It should be noted that while ultrasonication can effectively remove most air from superhydrophobic surfaces, some sub-micron-sized bubbles may remain at the solid–liquid interface. For clarity, samples with air trapped before ultrasonication treatment are labeled as ‘w/air’, while those after ultrasonication treatment are labeled as ‘w/o air’.

### Cell adhesion on various TiO_2_ nanostructures

We previously demonstrated that the number of adherent cells on the surface of SHB-NPA is not affected by the presence of air [43]. To investigate whether this holds true universally, we compared cell adhesion on various materials with water contact angle higher than 150°. Since ultrasonication only removes the air film layer at the solid–liquid interface without altering their nanostructure or chemical properties, the effect of air on cell adhesion can be evaluated by directly comparing the number and spreading state of cells on the material before and after air removal.

**Figure 3.**
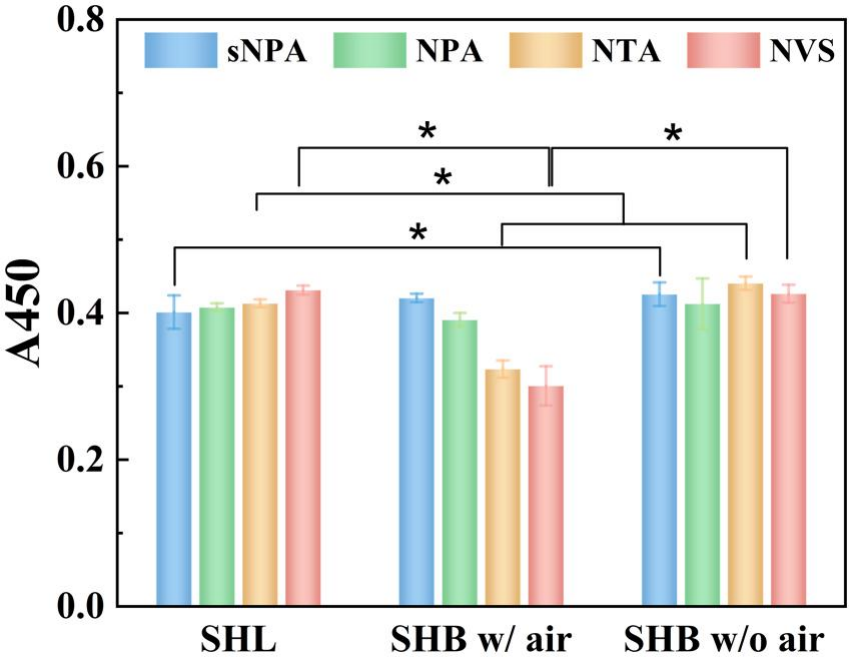
The number of adherent MC3T3-E1 cells after 2 h of culture on various samples. SHL refers to the as-prepared hydrophilic TiO_2_ nanostructures, and SHB refers to silanized hydrophobic counterparts. ‘w/o air’ and ‘w/air’ indicate that SHB materials were treated with and without ultrasonication, respectively. *P < 0.05.

The numbers of adherent cells on various sample surfaces was measured using the CCK-8 assay after 2 h (Figure 3). Numerous cells adhere to all four hydrophilic TiO_2_ nanomaterials, with no discernible differences, indicating that the morphology of the hydrophilic nanostructures does not significantly affect cell adhesion. For the SHB samples with trapped air, the number of adherent cells decreases as the nanostructure size increases. In the case of smaller nanostructures (sNPA and NPA), there is no significant difference in cell adhesion between the SHB material with air and the corresponding hydrophilic surface, consistent with our previous study [43]. However, for larger nanostructures (NTA and NVS), the number of adherent cells on the superhydrophobic surfaces with air is significantly lower than on the hydrophilic surfaces with the same morphology.

After the trapped air at the solid–liquid interface of the SHB samples was removed by ultrasonication, the differences in the number of adherent cells among the SHB samples with different structures disappeared. All surfaces exhibited high levels of cell adhesion, with no significant statistical differences. This suggests that, in the absence of trapped air, the morphology of the nanostructures does not significantly affect initial cell adhesion on SHB materials, similar to the hydrophilic counterparts. Surprisingly, the number of adherent cells on SHB materials without trapped air is not less than that on hydrophilic samples, with SHB-sNPA and SHB-NTA showing even slightly higher adhesion. Furthermore, after the removal of trapped air, the number of adherent cells on the SHB-NTA and SHB-NVS surfaces increases significantly compared to when air is present. These results suggest that, without trapped air, surface morphology of the hydrophobic TiO_2_ nanomaterials does not affect the number of adherent cells. However, as the nanostructure size increases, air content also increases, which further reduces cell adhesion. In other words, the size dimension of the nanostructure affects air content, and the combination of these factors influences cell adhesion on SHB materials with air.

**Figure 4.**
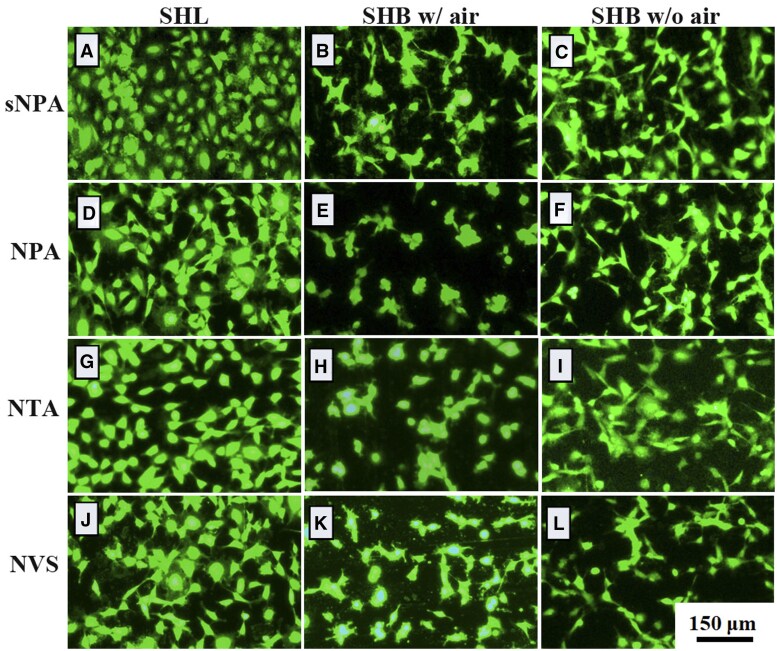
Fluorescence staining images of MC3T3-E1 cells after 2 h of adhesion on different sample surfaces with distinct nanostructures: (**A-C**) sNPA, (**D-F**) NPA, (**G-I**) NTA and (**J-L**) NVS, under SHL and SHB conditions with (w/) air and without (w/o) air.

To characterize cell spreading after 2 h of culture on various samples, MC3T3-E1 cells were stained with calcein AM and observed under a fluorescence microscope. As shown in Figure 4, a large number of cells adhered to and spread on all four SHL materials, with no significant differences in cell morphology or quantity. Among the SHB materials, only the cells on the sNPA sample exhibited partial spreading, while cells on the other samples remained clustered without spreading. After ultrasonication to remove trapped air, the adherent cells showed improved spreading, forming connections between cells. However, cell spreading on these surfaces remained limited, with more clustering observed compared to the SHL materials with the same microstructure.

To further investigate cell spreading, a scratching experiment was conducted on SHL-NTA and SHB-NTA samples. Cells on the SHB-NTA sample with trapped air remained clustered after 18 h culture, whereas cells on the SHB-TNA sample without air spread well (Supplementary Figure S1). After 6 h of culture following scratching, many cells near the edge of the scratched area migrated on the SHL-TNA sample, initiating scratch closure. In contrast, only a few cells on SHB-NTA began to migrate, regardless of the presence of trapped air. Herein, cell spreading was more pronounced on SHL materials, where cells adhered and spread uniformly, while SHB materials, especially those with trapped air, showed limited spreading with more clustering. Ultrasonication improved cell spreading on SHB surfaces, but migration remained restricted compared to SHL materials.

Since cell spreading on SHB materials is more limited, it is challenging to directly compare the number of adherent cells on SHB materials without trapped air to those on SHL materials due to cell aggregation and clustering (Figure 4, comparing row three to row one). Figure 3 demonstrates that when trapped air is removed, superhydrophobic samples modified with fluorosilane do not have fewer adherent cells than the corresponding SHL samples, and may even have more, as seen with the sNPA and NVS samples. The primary distinction between the corresponding SHL and SHB surfaces without trapped air is the presence of the fluorine group, suggesting that while the fluorine group on the SHB materials does not inhibit cell adhesion, it restricts cell spreading. As a result, some cells remain spherical on superhydrophobic surfaces without trapped air, with noticeable aggregation, particularly on the NVS sample.

### Bubble tracking

**Figure 5.**
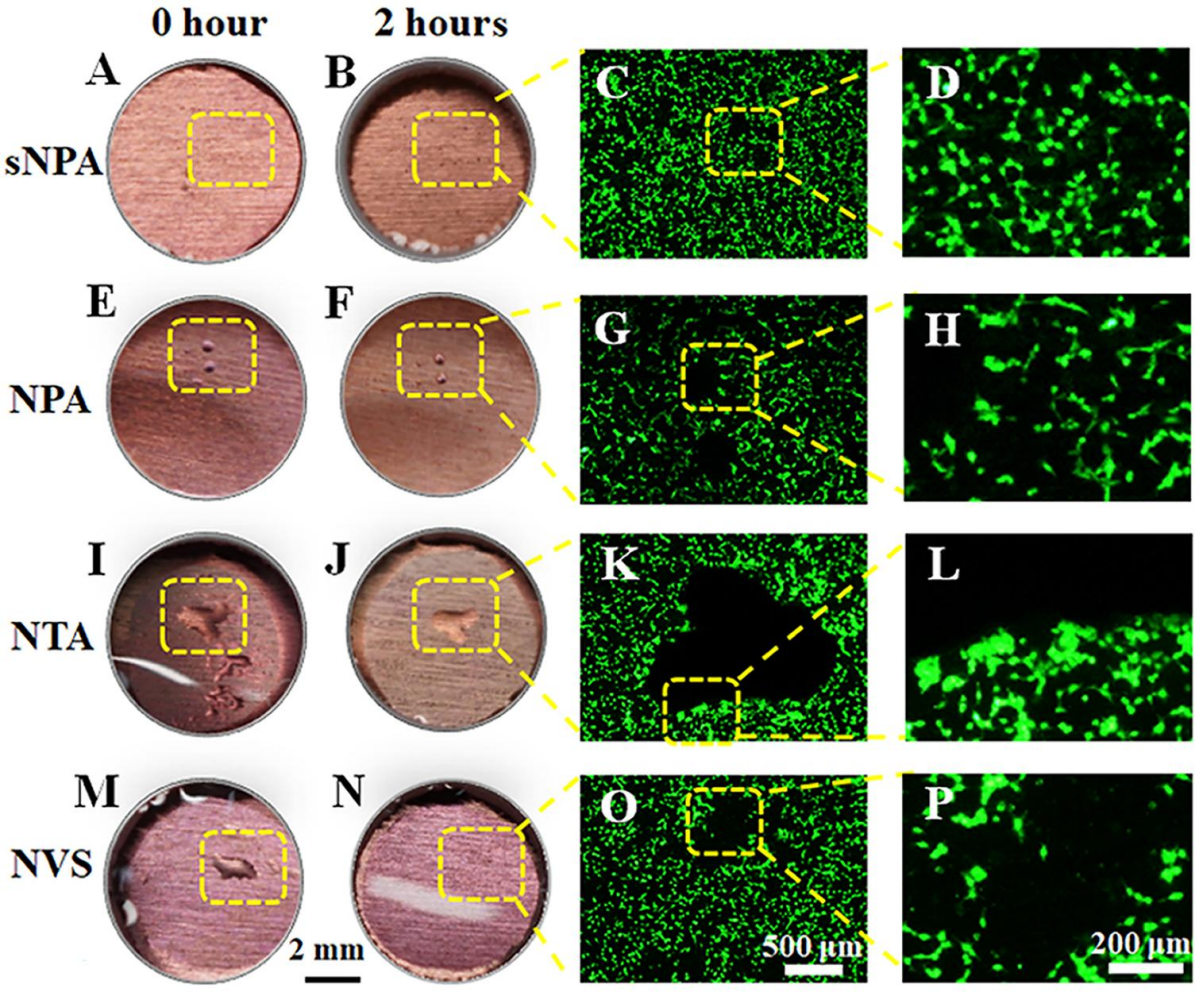
Optical images of bubbles over time and corresponding fluorescence images of MC3T3-E1 cells on different SHB samples with varying nanostructures: (**A-D**) sNPA, (**E-F**) NPA, (**I-L**) NTA, and (**M-P**) NVS.

It is worth noting that the bubbles formed when the surface of the SHB material comes into contact with the cell suspension vary in size. These bubbles can change size during the experiment and may even disappear from the material's surface. To investigate the role of these micron-sized bubbles in the cell adhesion process, the samples were labeled and the visible bubbles were compared before and after 2 h of incubation (Figure 5). Few observable bubbles (>0.1 mm) were present on the surface of the SHB-sNPA sample (with nanostructure size of about 20 nm) either at the start (Figure 5A) or after 2 h of culture (Figure 5B). Careful observation under a fluorescence microscope showed that cells were evenly distributed across the surface, with no distinct micropatterns in cell distribution (Figure 5C and D).

As the nanostructure size increased, bubbles ranging from 300 to 400 μm appeared on the superhydrophobic NPA sample (Figure 5E). These bubbles shrank slightly after 2 h of incubation (Figure 5F). Notably, no cells adhered to the regions containing trapped air, while cells accumulated in clusters around the bubbles, creating an oval-shaped cell-free zone (Figure 5G and H). The size and shape of this cell-free zone closely matched the air pockets visible in the optical images (Figure 5E and F). A similar phenomenon was observed on the superhydrophobic NTA sample (Figure 5I), where bubbles of different sizes merged and connected, forming a large bubble approximately 1–2 mm in size. The cell distribution around this bubble formed a distinctive patch (Figure 5K and L), corresponding to the bubble shape shown in Figure 5J.

The superhydrophobic NVS surface also traps micron-sized bubbles (Figure 5M), however, no silver mirror effect is observed, as shown in Figure 2H. This is because there are a lot of proteins in the cell culture medium, which not only reduce the interfacial tension of the solution, but also adsorb onto the surface of the superhydrophobic material, altering its properties. Therefore, air cannot accumulate in large quantities as it does at the pure water–superhydrophobic material interface. The micron-sized bubbles on the surface disappeared after 2 h of culture (Figure 5N). However, using a fluorescence microscope, we could still identify a cell-free patch where the air bubbles had been. This suggests that the initial presence of bubbles influences the sedimentation and distribution of the cell suspension, leading to altered cell patterns on the sample surface. These observations indicate that bubbles larger than 100 µm on the superhydrophobic surface hinder cell adhesion, leading to cell aggregation and settling along the bubble’s triple line during the adhesion process.

### Fabrication of transparent superhydrophobic PDMS

So far, we have demonstrated that trapped air larger than 100 µm can inhibit cell adhesion by preventing direct contact between the solid and liquid. However, one key question remains: When bubbles are much larger than cells, will all the cells accumulate at the solid–liquid–air interface around the bubbles, or will some remain on top of the bubbles at the liquid–air interface, or both? Unfortunately, due to the opacity of the titanium materials, it is difficult to observe this process directly. To address this, we designed a transparent superhydrophobic PDMS material to track air changes and corresponding cell movements.

Briefly, a silicon template was fabricated using photolithography (Supplementary Figure S2), and PDMS micropatterns were created by pouring PDMS onto the silicon template. While the PDMS micropatterns exhibited good hydrophobicity, they did not achieve superhydrophobicity. By further spin-coating silica nanoparticles, superhydrophobic PDMS samples with micro-nano structures were successfully obtained.

**Figure 6.**
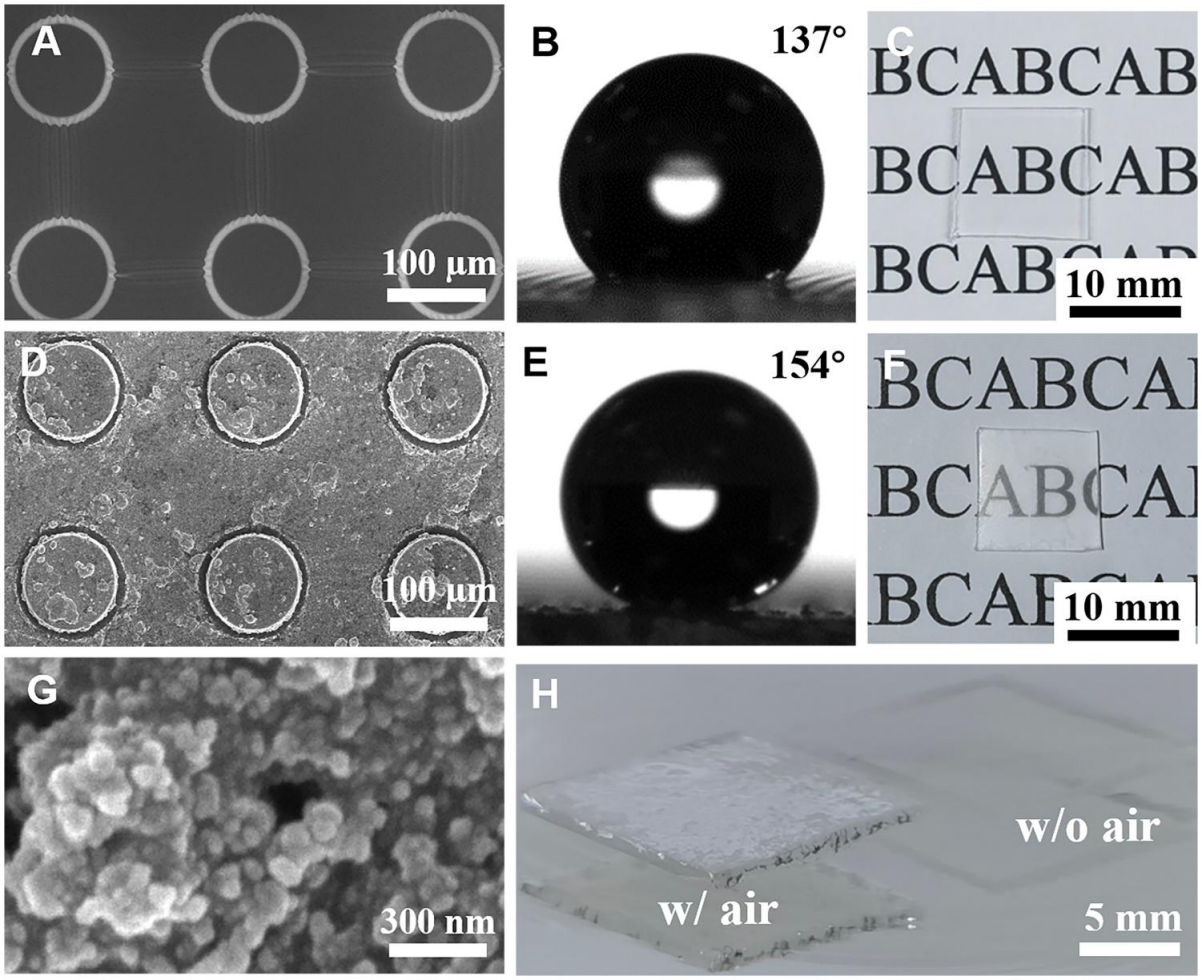
SEM images of the as-prepared PDMS micropattern (**A**) and SiO_2_-deposited PDMS with micro-nano structures (PDMS-MN) (**D**, **G**). Water contact angle images (**B**, **E**) and photographic images (**C**, **F**) of PDMS (**B**, **C**) and PDMS-MN (**E**, **F**). Optical images of PDMS-MN immersed in water before and after removing trapped air (**H**).

The surface of the as-prepared PDMS micropatterns feature a uniformly distributed micron-scale columnar structure with a diameter of 100 μm (Figure 6A). Its geometric parameters align with the micron-scale columnar groove structure of the silicon template surface, indicating that the PDMS successfully replicates the negative structure of the silicon template. The as-prepared PDMS micropatterns are hydrophobic, exhibiting a contact angle of approximately 137.26° ± 0.34° (Figure 6B). After the immobilization of SiO_2_ nanoparticles, a micro-nano structure forms on the PDMS surface (PDMS-MN, Figure 6D). During the curing process, the SiO_2_ nanoparticles adhere to the surface, with some particles aggregating into protrusions (Figure 6G). These irregular formations further increase surface roughness, enhancing the hydrophobicity to superhydrophobicity, with a contact angle of 153.87° ± 0.47° (Figure 6E).

The micron-sized PDMS (PDMS-MN) is transparent, with a transmittance of 98% (Figure 6C and Supplementary Figure S3). Although the deposition of silica reduces transmittance, the optimal amount still produces a superhydrophobic material with 85% transmittance (Figure 6F and Supplementary Figure S3). When PDMS-MN is immersed in water, numerous air bubbles become trapped at the solid–liquid interface (Figure 6H, ‘with air’). The air film causes specular reflection, giving the material a bright silver luster. Once the air is removed and the sample becomes fully wetted, the surface turns transparent, and the reflective silver layer disappears.

### Cell adhesion on superhydrophobic PDMS surface

**Figure 7.**
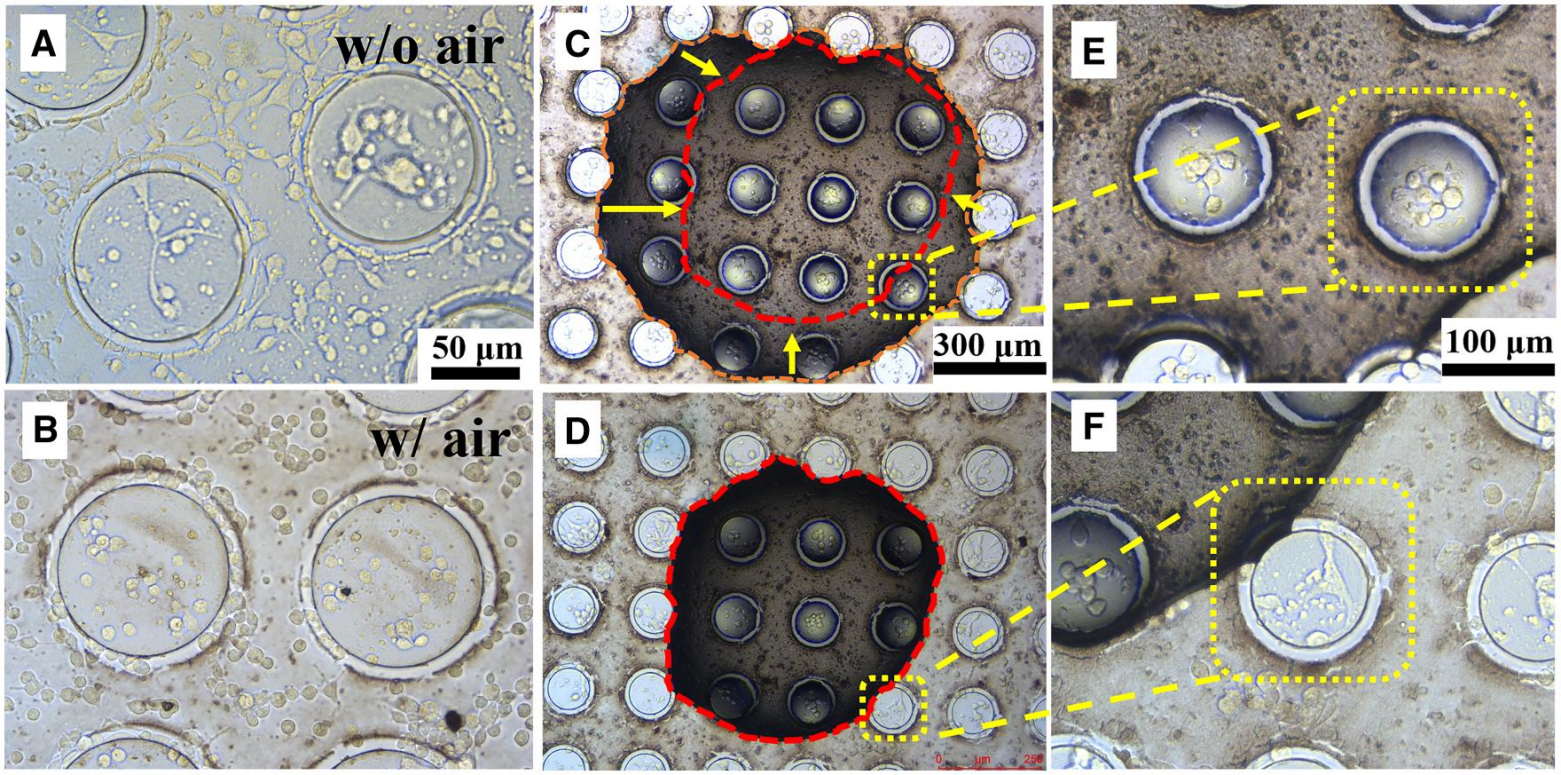
Morphology of MC3T3-E1 cells on superhydrophobic PDMS-MN in the absence (**A**) and presence (**B**) of trapped air. A large air bubble, on the scale of hundreds of microns, changes over the incubation time on the superhydrophobic PDMS-MN sample: (**C**) 2 h; (**D**) 4 h. The outer and inner irregular dashed circles represent the edges of air bubble after culturing for 2 and 4 h, respectively. (**E**) and (**F**) are magnified images of the dashed areas in (**C**) and (**D**), respectively.

To investigate the effect of the air layer on cell adhesion to the superhydrophobic MN-PDMS sample, we removed the trapped air from the solid–liquid interface and compared it with the sample that retained the air film layer. After 2 h of cell incubation on the superhydrophobic sample without air, some cells spread out and connected with surrounding cells (Figure 7A). In contrast, on the superhydrophobic sample with air, most cells remained spherical, showing no obvious pseudopodia or spreading (Figure 7B). This indicates that the trapped air on the superhydrophobic surface hinders cell attachment and spreading, consistent with results observed on superhydrophobic titanium samples.

Next, we examined a large air bubble in millimeter size and observed changes in the cells around the bubbles on the superhydrophobic PDMS-MN surface after 2 h (Figure 7C and E) and 4 h (Figure 7D and F) of incubation. After 2 h of culture, as shown in the area enclosed by the outer irregular dashed circle (approximately 0.6 mm^2^) where the air was, cells were primarily distributed on top of the cylindrical structures and remained in a spherical state. No cells were found in the gaps between the cylindrical structures. This suggests that air bubbles in millimeter size hinder the ‘infiltration’ of the cell suspension into the spaces between the cylinders, thus limiting cell contact and adhesion in these areas. Since the height of the columnar structures is about 16 μm and cells can ‘hover’ on the top without spreading, it is speculated that the height of the milimeter-sized bubbles slightly exceeds the height of the columns, preventing the cells from intimate contact with the column tops.

As the incubation time was extended to 4 h, the volume of the air bubbles decreased, as illustrated by the inner irregular dashed circle in Figure 7D. This shrinkage caused the air–liquid–solid three-phase contact line to move inward, reducing the area to approximately 0.4 mm^2^. The arrows in Figure 7C illustrate this process, which may be due to the dissolution of gas into the liquid. The columnar structures between the two irregular dashed circles were no longer covered by the air bubble, and the cells spread across the tops of the cylinders, similar to the cells outside the bubble from the beginning. In contrast, cells within the bubble area (Figure 7E) remained spherical. This is because the PDMS columns outside the air bubble had direct contact with cell suspension, allowing the cells to spread upon intimate contact with PDMS. This observation clearly demonstrates that millimeter-sized bubbles can inhibit cell adhesion and growth on the superhydrophobic PDMS surface. It is worth noting that, although the experiments on PDMS cannot be directly compared to those on titanium dioxide, they show that when a superhydrophobic material comes into contact with a cell solution, cells can remain suspended at the air–liquid interface if the trapped air bubbles between the solid and liquid are sufficiently large.

Hence, trapped air at the liquid–solid interface not only reduces the number of cells adhering to the surface of the superhydrophobic material, but also reduces the degree of spreading of cells on the surface of the material, resulting in a certain degree of agglomeration of cells. When ultrasonication is used to remove trapped air on the surface of the superhydrophobic sample, the adherent cell number increases significantly, connections are formed between cells and the degree of spreading is also improved. It is worth noting that there is no significant difference in the adherent cell number or the spreading degree of cells when comparing four superhydrophobic materials without trapped air, indicating that nanostructure sizes have no significant impact on cell attachment in the absence of air, similar to that on SHL counterparts. In addition, the cells adhered to four different nanostructured SHL materials have similar morphologies, showing fibrous spreading.

### Phenomenological model

**Figure 8.**
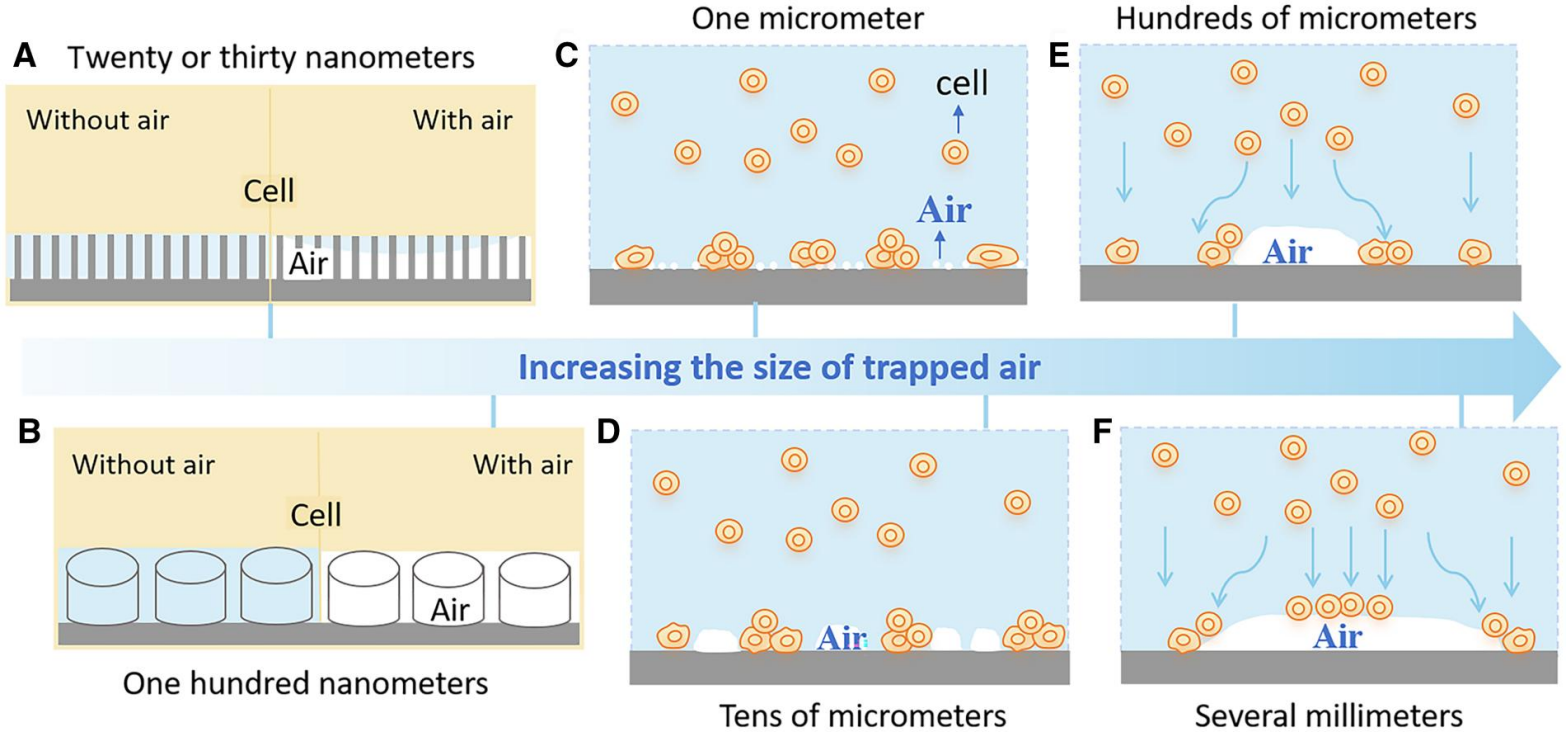
Phenomenological model illustrating the size effect of surface structure and trapped air on mammalian cell adhesion on materials with varying trapped air size: (**A**) less than 30 nanometers, (**B**) approximately 100 nanometers, (**C**) about 1 micrometer, (**D**) about tens of micrometers, (**E**) hundreds of micrometers, and (**F**) several millimeters.

Generally speaking, the effect of superhydrophobic materials on mammalian cell adhesion is influenced by the micro-nano size of the material. Specifically, larger nanostructures trap more air, which more severely inhibits cell adhesion. For SHL or SHB TiO_2_ samples with no air on the surface—i.e. complete wetting—cells can adhere and spread well (Figure 8A and B). However, the hydrophobic groups on the surface hinder full cell spreading, leading to increased aggregation.

In the case of superhydrophobic materials, when the nanostructure size is as small as 20 or 30 nm, the size of the air bubbles is comparable to that of the nanostructures (Figure 8A). These air bubbles, being only tens of nanometers in size, are very small relative to cells that are tens of microns in size, allowing the cells to contact the top of the nanostructure. As a result, the presence of bubbles does not reduce the number of adherent cells.

When the size of the nanostructure increases to 100 nm (Figure 8B), the air bubbles also grow and can connect to form larger bubbles, ranging from hundreds of nanometers to millimeters. When the bubble size remains small relative to cell size—specifically, less than 1 μm—cells can still come into contact with the nanomaterials and adhere (Figure 8C). Even when the bubble size is comparable to that of the cells, adherence can still occur in the absence of large bubbles. When the bubble size is similar to or smaller than that of the cell, gravity can cause the cell to fall to the solid surface and adhere (Figure 8D).

However, when the bubble size becomes significantly larger than that of the cells, cells located above the air bubble may slide along its surface or around it due to gravity, while some may remain above the bubble (Figure 8E). As the bubble size continues to increase to hundreds of microns, more cells can stay above it (Figure 8F). As illustrated in Figure 7D, as the air bubble decreases in size, cells originally above the bubble can settle to the solid–liquid interface and adhere. Therefore, the size of the nanostructure ultimately determines the size of the bubbles, which further affects cell adhesion on the surface of superhydrophobic materials.

Overall, the significant inhibition of cell adhesion by superhydrophobic materials results from the combined effects of nanostructure and trapped air. It is worth noting that cell adhesion is a complex process influenced by multiple factors, including surface charge, cell type and protein composition. For example, at lower cell densities, the phenomenon observed in Figure 5 is less pronounced due to the sparse distribution of cells. Herein, a more comprehensive investigation incorporating these additional factors would provide deeper insights for future studies.

## Conclusion

In summary, the influence of morphology and trapped air on cell adhesion to materials exhibiting extreme wetting properties was systematically explored. The results show that cell adhesion on all four SHL TiO_2_ samples was high. The small SHB-sNPA and NPA samples had comparable cell adhesion numbers to their SHL counterparts. As the size of the SHB nanostructures increased, the air content at the sample interface also increased, leading to a decrease in the number of adhered cells. When air was removed by ultrasonication, all SHB samples showed cell adhesion numbers comparable to those of their corresponding SHL samples. Notably, cells adhered to the SHB surfaces of the sNPA and NTA samples in even greater numbers than to their corresponding SHL samples with the same morphology. Cells on SHB materials with trapped air remained mostly spherical and tended to aggregate, whereas cells on SHB materials without air spread out more, although they still formed clusters compared to their SHL counterparts. Further tracking of bubbles on the surface of the SHB samples revealed that no cells adhered to areas occupied by micron-sized air, and more cells gathered along the solid–liquid–gas triple line. Observations of particularly large bubbles on the SHB PDMS surface showed that cells could gather at the air–liquid interface and adhere to the material surface.

Future designs of biomedical materials or devices incorporating superhydrophobicity should focus on tailoring the surface nanostructure to optimize performance. This approach enables the creation of surfaces that can precisely regulate cellular responses, with potential applications in anti-biofouling coatings, tissue scaffolds and vascular implants.

## Supplementary Material

rbaf021_Supplementary_Data
