# Supplementary material for: Dimensional effects of surface morphology and trapped air on mammalian cell adhesion to special wetting surfaces
Source: Regen Biomater. 2025 Apr 1;12:rbaf021. doi: 10.1093/rb/rbaf021 (PMC12017620; doi:10.1093/rb/rbaf021)
Supplement: rbaf021_Supplementary_Data [file rbaf021_supplementary_data.docx]

**Supporting information**

**Dimensional effects of surface morphology and trapped air on mammalian cell adhesion to special wetting surfaces**

*Zhiwei Chen^a,1^, Yun Yang^a,b,1^, Shaohua Xu^a,b^, Zhenyu Shen^a^, Yijian Tang^a^,* *Yisheng Lin^a^ and Qiaoling Huang^a,b,*^*

1. Research Institute for Biomimetics and Soft Matter, Fujian Provincial Key Laboratory for Soft Functional Materials Research, Department of Physics, College of Physical Science and Technology, Xiamen University, Xiamen 361005, China.
2. Jiujiang Research Institute of Xiamen University, Jiujiang, 332000, China

^1^ These authors have contributed equally to this work.

* Corresponding author. Email address: [qlhuang@xmu.edu.cn](mailto:qlhuang@xmu.edu.cn) (Q. Huang)

KEYWORDS: Superhydrophobic materials, Mammalian cell adhesion, Trapped air, Dimensional effects


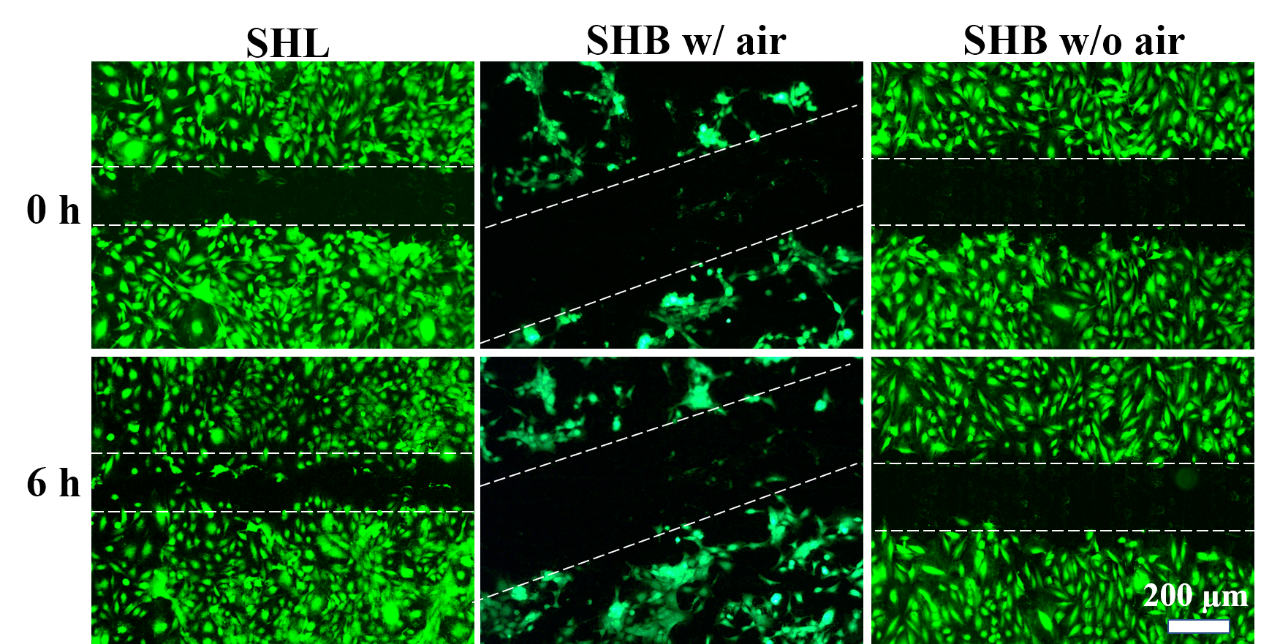


**Figure S1.** Cell motility on SHL-NTA and SHB-NTA samples assessed by a wound healing assay. A scratch was created using a 10 µL pipette tip on the confluent cell layer, and its closure was monitored after 6 hours.


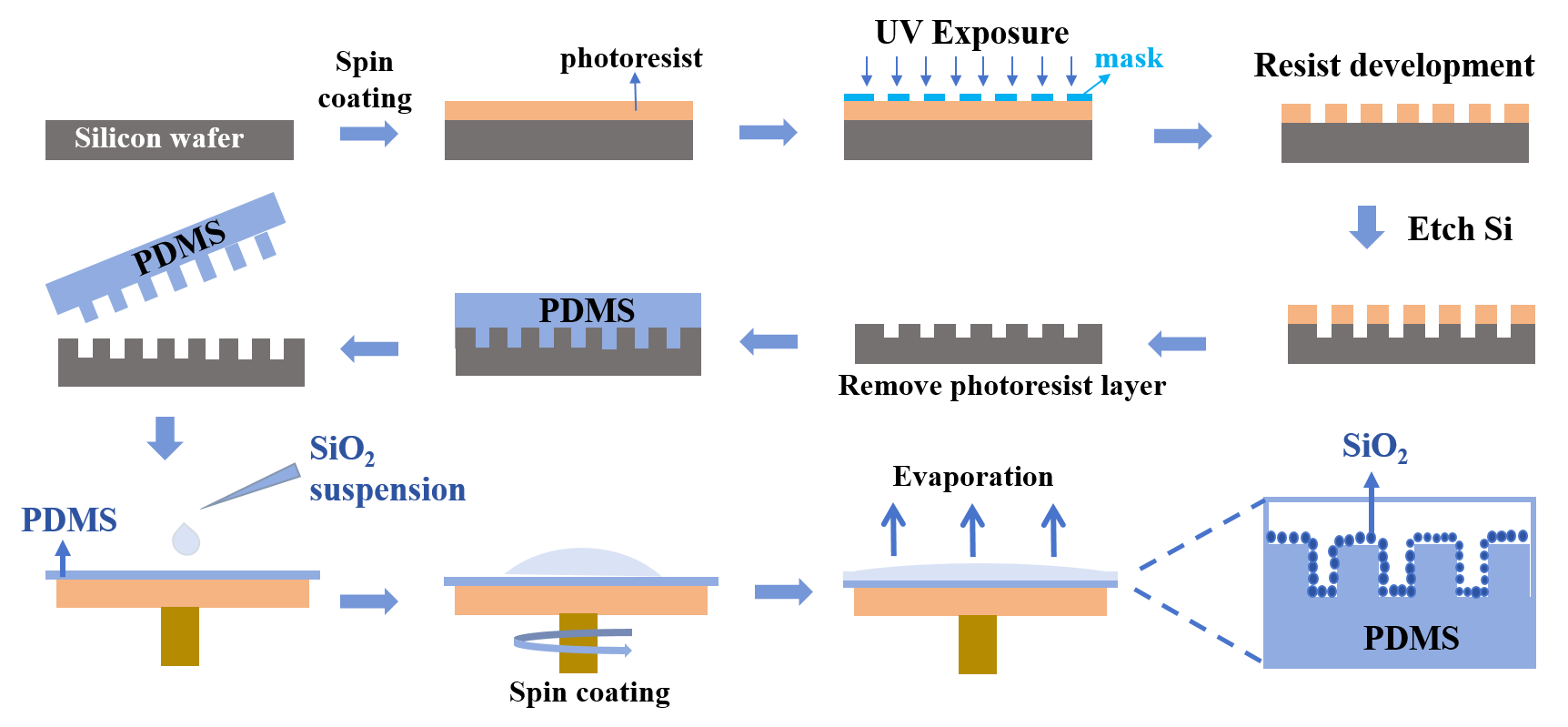


**Figure S2.** Schematic diagram of silicon micropattern fabrication using photolithography.

**Figure S3.** Transmittance spectra of PDMS and PDMS-MN samples.
